# Cardiac toxicity of trastuzumab in metastatic breast cancer patients previously treated with high-dose chemotherapy: a retrospective study

**DOI:** 10.1038/sj.bjc.6603060

**Published:** 2006-03-28

**Authors:** C Bengala, C Zamagni, P Pedrazzoli, P Matteucci, A Ballestrero, G Da Prada, M Martino, G Rosti, M Danova, M Bregni, G Jovic, V Guarneri, M Maur, P F Conte

**Affiliations:** 1 Division of Medical Oncology, University Hospital, Via del Pozzo, 71-41100 Modena, Italy; 2 Division of Medical Oncology, S. Orsola-Malpighi Hospital, Bologna, Italy; 3 Division of Medical Oncology, Niguarda Hospital, Milan, Italy; 4 Division of Medical Oncology, National Cancer Institute, Milan, Italy; 5 S. Martino University Hospital, Genoa, Italy; 6 Division of Medical Oncology, Maugeri Cancer Foundation, Pavia, Italy; 7 Bone Marrow Transplantation Unit, Civic Hospital, Reggio Calabria, Italy; 8 Division of Medical Oncology, Civic Hospital, Ravenna, Italy; 9 Division of Medical Oncology, University Hospital, Pavia, Italy; 10 Bone Marrow Transplantation Unit, S. Raffaele Hospital, Milan, Italy

**Keywords:** trastuzumab, cardiac toxicity, metastatic breast cancer, high-dose chemotherapy

## Abstract

HER-2 overexpression is associated to a poor prognosis in high-risk and metastatic breast cancer (MBC) patients treated with high-dose chemotherapy (HDC). HER-2 status is also a predictive factor and when trastuzumab is administered in combination with or sequentially to chemotherapy, a significant disease-free and/or overall survival improvement has been observed in HER-2+ early and MBC. Unfortunately, in both settings, trastuzumab is associated with an increased risk of cardiac dysfunction (CD). We have reviewed the clinical charts of HER-2-overexpressing MBC patients treated with trastuzumab after HDC. Age, baseline left ventricular ejection fraction (LVEF), radiation therapy on cardiac area, exposure to anthracycline, single or multiple transplant, high-dose agents, trastuzumab treatment duration were recorded as potential risk factors. In total, 53 patients have been included in the analysis. Median LVEF at baseline was 60.5%; at the end of trastuzumab (data available for 28 patients only), it was 55% (*P*=0.01). Five out of the 28 (17.9%) patients experienced CD. Two out of 53 (3.8%) patients developed a congestive heart failure. Age ⩾50 years and multiple transplant procedure were potential risk factors for CD. The overall incidence of CD observed in this population of HER-2+ MBC patients treated with trastuzumab after HDC is not superior to that reported with concomitant trastuzumab and anthracyclines. However, patients with age ⩾50 years or receiving multiple course of HDC should be considered at risk for CD.

High-dose chemotherapy with autologous hemopoietic progenitor support has been compared to standard dose chemotherapy in high-risk early breast cancer as well as in metastatic disease. In high-risk breast cancer, there are negative trials showing no superiority for high-dose chemotherapy (Bergh *et al*, 2000; Hortobagyi *et al*, 2000; Tallman *et al*, 2003); in contrast, other trials have shown significant improvement in relapse-free and overall survival (Roche *et al*, 2003; Rodenhuis *et al*, 2003; Nitz *et al*, 2005). In metastatic breast cancer, whereas some trials have failed to demonstrate a survival benefit, others have suggested an advantage in event-free survival in favour of high-dose treatment (Stadtmauer *et al*, 2000; Crown *et al*, 2004; Lotz *et al*, 2005; Schmid *et al*, 2005). In spite of these conflicting results, it is however clear that, at present, high-dose chemotherapy cannot be considered standard treatment for any breast cancer patient. On the other side, longer follow-up of the randomized trials and a meta-analysis of their pooled data might confirm a limited advantage; moreover, because of the reduced treatment-related mortality and availability of new anticancer drugs, this treatment modality could still represent a field of research for some subsets of patients.

It is now clear that breast cancer represents a very heterogeneous disease and, from a clinical stand point, at least three breast cancer subtypes must be recognized: hormone-receptor positive, HER-2 positive and triple negative. In particular, overexpression of HER-2 oncogene occurs in 20–30% of the patients with invasive breast cancer and is associated with poor prognosis and decreased overall survival (Slamon *et al*, 1987). Retrospective studies have shown that HER-2 overexpression is an independent poor predictive factor of outcome after high-dose chemotherapy both in high-risk and metastatic breast cancer (Bitran *et al*, 1996; Kim *et al*, 2001; Nieto *et al*, 2002; Guarneri *et al*, 2004). Moreover, Rodenhuis *et al* (2003) in the National Duch trial of high-dose chemotherapy in high-risk breast cancer have reported an unplanned subset analysis based on HER-2 status suggesting that the improvement of relapse-free survival of high-dose chemotherapy in patients with stage II and III breast cancer and 10 or more axillary nodes may be confined to patients with HER-2-negative tumours. A part from its possible prognostic and predictive role, the HER-2 status determines the choice of treatment: when trastuzumab, a humanised monoclonal antibody that selectively binds to the extracellular domain of the HER-2 protein, is used in combination with chemotherapy, response rate, time to progression and median overall survival are improved (Slamon *et al*, 2001; Marty *et al*, 2005). Unfortunately, Trastuzumab was unexpectedly found to be associated with an increased risk of cardiac dysfunctions and asymptomatic decrease in left ventricular ejection fraction (LVEF); this risk was especially high in patient receiving concurrent anthracyclines, older than 50 years or with decreased LVEF after anthracyclines (Seidman *et al*, 2002; Perez and Rodeheffer, 2004; Tan-Chiu *et al*, 2005).

In order to estimate the cardiac tolerability of trastuzumab after high-dose chemotherapy, we have retrospective analysed the clinical files of HER-2-positive breast cancer patients treated with high-dose chemotherapy in the Italian Centers of the GITMO group (Gruppo Italiano Trapianto Midollo Osseo).

## PATIENTS AND METHODS

A questionnaire was sent to 11 major Italian centres. The following information were requested: histology, hormonal receptor status, HER-2 status (IHC or, if available, FISH status), radiation therapy on cardiac area, previous chemotherapy with regimes including anthracycline and paclitaxel, cumulative dose of anthracycline, use of cardioprotective agents, status of disease at the time of high-dose chemotherapy procedure, single or multiple transplant procedure, dose of cyclophosphamide >4 g m^−2^ and mitoxantrone >60 mg m^−2^, status of disease after transplant, dose and schedule of trastuzumab, cytotoxic agents associated with trastuzumab, LVEF before starting trastuzumab and at the end of treatment, any episode of congestive heart failure (CHF). Cardiac dysfunction was defined as (1) a decline of LVEF ⩾10% to below 50%, (2) a decline of LVEF between 5 and 9% to below 50% with symptomatic (NYHA class III–IV) CHF, (3) any symptomatic (NYHA class III–IV) CHF event. Any one of the three criteria was sufficient to confirm a diagnosis of cardiac dysfunction.

### Statistical analysis

The Wilcoxon matched-pairs signed-ranks test was used to determine if the distribution of the LVEF at baseline and at the end of treatment was the same. Time to suspension and time to cardiac event were calculated using the Kaplan–Meier method. Time to suspension was calculated from the date of beginning of the treatment with trastuzumab until the suspension or last follow-up. Time to cardiac event was calculated from the date of beginning of the treatment with trastuzumab until the date of the cardiac event or last follow-up. The log-rank test was used to compare the curves of time to cardiac event. The hazard ratio and the 95% CI was calculated. The level of significance was set to be 0.05. The statistical software used for the analysis was STATA/SE version 8.0.

## RESULTS

All the centres returned the questionnaire and we could collect data from 53 patients treated with trastuzumab for metastatic disease between February 1999 and June 2005.

Patients characteristics are shown in Table 1. Median age was 47 years (range 29–66). Histology type was ductal carcinoma in 85% of the patients; hormone receptor status was positive in 44.2% of the patients. HER-2 expression was scored by immunohistochemistry method as 3+ in 49 patients and as 2+ in four patients; in the 2+ patients, HER-2 amplification was confirmed by FISH. A total of 44 patients (83%) had received treatment with anthracycline/paclitaxel-containing regimens and 16 patients (30%) had received radiation therapy on cardiac area. Median cumulative doses of doxorubicin or epidoxorubicin were 266 mg m^−2^ (range 60–500) and 395 mg m^−2^ (range 120–660), respectively. High-dose chemotherapy was administered as adjuvant treatment to 17 patients, for locally advanced disease to three patients and for metastatic disease to 33 patients. A total of 21 patients received a single course of high-dose chemotherapy and 32 patients a double course of high-dose chemotherapy. High-dose therapy included melphalan–thiotepa in 27 patients (50.9%), mitoxantrone–melphalan in 14 patients (26.4%), mitoxantrone–thiotepa in six patients (11.3%), mitoxantrone–melphalan–thiotepa in three patients (5.7%); three patients (5.7%) received other regimens. Median interval between high-dose chemotherapy and the beginning of trastuzumab was 6 months (range 0–90). Trastuzumab was administered at weekly or 3-weekly schedule in 33 and 20 patients, respectively. Trastuzumab was given as single agent in 17 patients, in combination with paclitaxel in 21 patients, and with vinorelbine in 15 patients. Median duration of treatment with trastuzumab was 11 months (1–46) with 32 patients receiving trastuzumab for more than 12 months. At the time of analysis, 41 patients have stopped the treatment with trastuzumab: seven for cardiac toxicity, 29 for progression of disease, four for patient's decision, one patient died early after the administration of trastuzumab; 12 patients are still on treatment.

### Cardiac dysfunction

**Figure 1:**
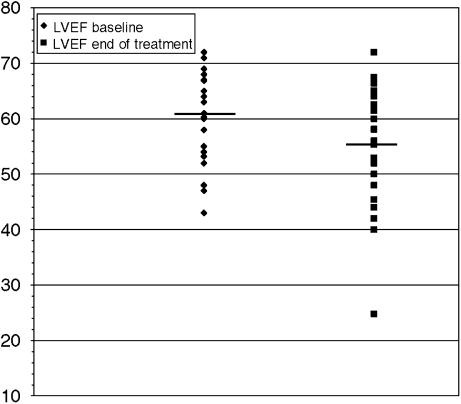
Values and median of LVEF at baseline and at the end of treatment with trastuzumab.

Baseline LVEF was available for all patients; LVEF at the end of treatment was available for 28 patients and 13 patients had also a third measurement of LVEF. Median LVEF values at baseline and at the end of trastuzumab were 60.5% (43–72) and 55% (25–72), respectively (Figure 1); this difference resulted statistically significant with a *P*-value of 0.01. At the end of treatment with trastuzumab, seven patients (25%) had a reduction of LVEF to less than 50%. In this group of patients, one patient had a decline of LVEF of 3% without cardiac symptoms, one patients had a reduction of LVEF of 7% and developed mild cardiac symptoms classified as NYHA class II, five patients had a decline of LVEF ⩾10% and two of these developed congestive heart failure classified as NYHA class III. According to our definition, five out of 28 patients with evaluable LVEF experienced a cardiac event (17.9%).

On the entire group of patients, two out of 53 (3.8%) developed a congestive heart failure (NYHA class III). Both patients had a decline of LVEF >10% to below 50%. These two patients started trastuzumab within 2 months from high-dose chemotherapy and the duration of treatment with trastuzumab was 18 and 46 months, respectively. One of these two patients had previously received doxorubicin (cumulative dose of 300 mg m^−2^).

Trastuzumab was discontinued in seven patients with a LVEF<50%. Follow-up data for cardiac function recovery are available for four patients only: all these patients recovered a normal LVEF at a median interval of 11 months (range 3–19).

### Analysis of risk factors

The incidence of cardiac dysfunction was analysed according to risk factors including age, baseline LVEF, previous radiation therapy on cardiac area, single *vs* multiple transplant, dose of cyclophosphamide and mitoxantrone, previous anthracycline exposure and duration of treatment with Trastuzumab (Table 2). Age ⩾50 years was the only factor significantly associated with cardiac dysfunction (*P*: 0.027). Moreover, we have analysed risk factors associated with a significant decline of LVEF at the end of treatment with trastuzumab (Table 3). Age ⩾50 years and multiple courses of high-dose chemotherapy were associated with a 13% reduction in LVEF (*P*=0.02) and a 4% reduction in LVEF (*P*=0.02), respectively.

Baseline LVEF<55%, previous treatment with anthracicline/paclitaxel, high-dose of cyclophospamide alone or in combination with high-dose mitoxantrone and duration of treatment with trastuzumab >12 months were not associated with a significant reduction of LVEF. Previous radiation therapy on cardiac area was associated with a 4% reduction of LVEF, but this value did not reach the statistical significance (0.08).

## DISCUSSION

Several studies have shown that high-dose chemotherapy is not able to modify the poorer prognosis of HER-2-positive breast cancer patients (Slamon *et al*, 1987; Bitran *et al*, 1996; Nieto *et al*, 2002; Rodenhuis *et al*, 2003; Guarneri *et al*, 2004). It is therefore clear that these patients should be treated with trastuzumab. Unfortunately, very few data on the cardiac tolerability of trastuzumab after high-dose chemotherapy are available.

A retrospective analysis on 1219 patients treated for metastatic disease with standard chemotherapy with or without trastuzumab in seven phase II and III clinical trials showed symptomatic cardiac dysfunction (NYHA class III–IV) in 2–16% of patients who received trastuzumab. The incidence was 2–4% for patients receiving first-line trastuzumab as single agent or in combination with paclitaxel, while it increased to 16% for patients receiving trastuzumab plus doxorubicin–cyclophosphamide combination. The overall cardiac dysfunction rate was 13 and 27% for patients receiving trastuzumab plus paclitaxel or trastuzumab plus doxorubicin–cyclophosphamide (AC) combination, respectively. Analysis of potential risk factors for cardiac dysfunction in patients with metastatic breast cancer treated with standard-dose chemotherapy plus trastuzumab has demonstrated that increasing age and the association of trastuzumab plus anthracycline was a statistically significant predictive factor. Other factors such as prior radiotherapy, and mean cumulative dose of doxorubicin were not found to be independent predictive factors of cardiac dysfunction (Seidman *et al*, 2002). Moreover, Perez and Rodeheffer (2004) reviewed data from new prospective clinical trials reporting lower incidence of trastuzumab-related cardiac toxicity when more stringent patient selection and cardiac monitoring criteria were adopted. More recently, Tan-Chiu *et al* (2005) reported data on cardiac dysfunction associated to herceptin in combination with paclitaxel following AC regimen in high-risk breast cancer. The incidence of CHF was 4.1%. Age ⩾50 years and baseline LVEF<55% were found as risk factors significantly associated with CHF.

Cardiotoxicity is also a major concern in the treatment with high-dose chemotherapy. Risk factors for cardiotoxicity include high-dose cyclophosphamide, high-dose mitoxantrone and previous radiation therapy on cardiac area (Morandi *et al*, 2005). Several studies failed to demonstrated a relationship between previous anthracycline exposure and development of cardiac toxicity following high-dose chemotherapy (Nieto *et al*, 2000; Petros *et al*, 2002). However, we and others have reported that high-dose alkylating agents in metastatic breast cancer patients previously exposed to cumulative dose of epirubicin ⩾450 mg m^−2^ is associated with a 4–5% of congestive heart failure rate (Alidina *et al*, 1999; Gennari *et al*, 1999; Bengala *et al*, 2001). Moreover, we have shown that a double course of high-dose chemotherapy after anthracycline-containing regimen in patients of metastatic breast cancer can induce a transient decline of LVEF to less than 50% in 14.3% of the patients (Bengala *et al*, 2003).

Our retrospective analysis shows that median LVEF is significantly lower after trastuzumab in patients previously treated with high-dose chemotherapy; moreover, in these patients, cardiac dysfunction rate and symptomatic cardiac failure (NYHA class III) rate were 17.9 and 3.8%, respectively. The incidence of CD was higher than that reported with trastuzumab as single agent or in combination with paclitaxel but not superior to that observed with trastuzumab administered concomitantly with anthracyclines. Age >50 years and multiple transplant procedures have been the only factors associated with an increased risk of cardiotoxicity. Interestingly, 83% of our patients had received anthracycline–taxane chemotherapy, another potentially cardiotoxic regimen, before HD treatment; moreover, duration of trastuzumab treatment was relatively long (median 11 months), with 32 patients treated for more than 1 year.

Nieto *et al* (2004) and Goncalves *et al* (2005) have prospectively studied the feasibility of the concurrent administration of trastuzumab with alkylator-based high-dose chemotherapy in breast cancer. End points of the studies were safety and pharmacokinetic profile evaluation. Trastuzumab was administered for a median of 9 weeks (range 9–48) and 15 weeks (range 1–39) in the two trials with no apparent increased toxicity and in particular cardiac dysfunction.

In conclusion, our retrospective analysis shows that the administration of trastuzumab after high-dose chemotherapy is feasible with an incidence of cardiac dysfunction and symptomatic cardiac failure not superior to that reported with trastuzumab in combination with anthracyclines. Age ⩾50 years, multiple courses of high-dose chemotherapy and prior radiation therapy on cardiac area are risk factors for cardiac dysfunction in this patient population.

## Figures and Tables

**Table 1:** Characteristics of the patients

	**47 years (29–66)**	
**Age: median (range)**	**No. of patients**	**%**
*Histology type*		
Ductal carcinoma	45	85
Lobular carcinoma	8	15

*Hormone receptor status*		
ER and/or PgR+	23	43.4

*HER-2 status*		
2+ (FISH positive)	4	7.5
3+	49	92.5

Radiation therapy on cardiac area	16	30.2
Prior anthracycline/paclitaxel	44	83.0

*HDCT*		
Adjuvant treatment	17	32.1
For metastatic disease	33	62.3
For locally advanced disease	3	5.7

Single course of HDCT	21	39.6
Multiple courses of HDCT	32	60.4
HDCT with CTX>4 g m^−2^	37	69.8
HDCT with DHAD >60 mg m^−2^	16	30.2

*Dose of trastuzumab*		
6 mg kg^−1^ 3 weekly^−1^	20	37.7
2 mg^−1^kg weekly^−1^	33	62.3

*Trastuzumab*		
Single agent	17	32.1
With paclitaxel	21	39.6
With vinorelbine	15	28.3

CTX=cyclophosphamide; DHAD=mitoxantron; ER=estrogen receptor; PgR=progesteron receptor; FISH=fluorescence *in situ* hybridisation.

**Table 2:** Potential risk factors for cardiac dysfunction (28 patients, five events)

	**No. of patients**	**No. of events**	**HR (95% CI)**	***P*-value**
*RT on cardiac area*				
No	18	3		
Yes	10	2	0.68 (0.07;6.7)	0.74

*HDCT*				
Single	8	1		
Multiple	20	4	1.22 (0.08;7.99)	0.87

*High-dose agents*				
No CTX-no DHAD	4	1		
CTX	13	1	0.19 (0.01;3.25)	0.45
CTX+DHAD	11	3	0.47 (0.04;6.23)	

*Anthracicline*				
No	4	2		
Yes	24	3	0.52 (0.05;5.05)	0.56

*Trastuzumab*				
⩽12 months	17	2		
>12months	11	3	0.39 (0.03;4.58)	0.43

*Age (years)*				
<50	19	1		
⩾50	9	4	8.79(0.88;88.08)	0.03

*LVEF baseline%*				
⩾55	21	4		
<55	7	1	0.71 (0.07;6.91)	0.76

CTX=cyclophosphamide; DHAD=mitoxantron; LVEF=left ventricular ejection fraction; HR=hazard ratio.

*P-*value: log rank test for time to cardiac dysfunction.

RT=radiation therapy.

**Table 3:** Potential risk factors for reduction of LVEF after treatment with trastuzumab

	**LVEF baseline median (range)**	**LVEF end of treatment median (range)**	***P*-value**
	60.5 (43–72)	55.05 (24.8–72)	0.03
*RT on cardiac area*			
Yes	62 (43–69)	52.5 (40–72)	0.08
No	60 (48–72)	61 (24.8–66)	0.18

*HDCT*			
Single	60.5 (47–65)	60 (40–66)	0.83
Multiple	60.5 (43–72)	56.8 (24.8–72)	0.02

*High-dose agents*			
No CTX,No DHAD	60.5 (53.2–64)	61 (24.8–66)	0.85
CTX	60 (43–72)	55.5 (45–72)	0.15
CTX+DHAD	60 (47–71)	58.1 (40–65)	0.11

*Anthracycline*			
Yes	60 (43–72)	59.05 (24.8–72)	0.10
No	64 (61–71)	53.5 (40–65.4)	0.14

*Trastuzumab*			
⩽12 months	60 (43–71)	58.1 (24.8–72)	0.31
>12months	61 (47–72)	60 (40–66)	0.07

*Age (years)*			
<50	60 (43–72)	60 (40–66)	0.49
⩾50	65 (53.2–71)	52 (24.8–72)	0.02

*LVEF baseline*%			
<55	52 (43–54)	45 (24.8–60)	0.73
⩾55	64 (55–72)	62 (40–72)	0.48

CTX=cyclophosphamide; DHAD=mitoxantron; LVEF=left ventricular ejection fraction.

*P-*value: Wilcoxon sign rank test. RT=radiation therapy.
